# Transcriptome analysis of *Corvus splendens* reveals a repertoire of antimicrobial peptides

**DOI:** 10.1038/s41598-023-45875-w

**Published:** 2023-10-31

**Authors:** Shalini Kannoth, Nemat Ali, Ganesh K. Prasanth, Kumar Arvind, Mohamed Mohany, Preety Sweta Hembrom, Shemmy Sadanandan, Deepa Azhchath Vasu, Tony Grace

**Affiliations:** 1 Department of Genomic Science, School of Biological Sciences, Central University of Kerala, Kasaragod, Kerala India; 2 Department of Biochemistry and Molecular Biology, School of Biological Sciences, Central University of Kerala, Kasaragod, Kerala India; 3 Department of Pharmacology and Toxicology, College of Pharmacy, King Saud University, Riyadh, 11451 Saudi Arabia; 4 Neurogenetics Branch, National Institute of Neurological Disorder and Stroke, National Institute of Health, Bethesda, MD 20892 USA

**Keywords:** Biotechnology, Drug discovery, Microbiology, Molecular biology, Zoology

## Abstract

Multidrug resistance has become a global health problem associated with high morbidity and mortality. Antimicrobial peptides have been acknowledged as potential leads for prospective anti-infectives. Owing to their scavenging lifestyle, *Corvus splendens* is thought to have developed robust immunity to pathogens found in their diet, implying that they have evolved mechanisms to resist infection. In the current study, the transcriptome of *C. splendens* was sequenced, and de novo assembled to identify the presence of antimicrobial peptide genes. 72.09 million high-quality clean reads were obtained which were then de novo assembled into 3,43,503 transcripts and 74,958 unigenes. About 37,559 unigenes were successfully annotated using SwissProt, Pfam, GO, and KEGG databases. A search against APD3, CAMP_R3_ and LAMP databases identified 63 AMP candidates belonging to more than 20 diverse families and functional classes. mRNA of AvBD-2, AvBD-13 and CATH-2 were found to be differentially expressed between the three tested crows as well as among the tissues. We also characterized *Corvus* Cathelicidin 2 (CATH-2) to gain knowledge of its antimicrobial mechanisms. The CD spectroscopy of synthesized mature *Corvus* CATH-2 peptide displayed an amphipathic α-helical structure. Though the synthetic CATH-2 caused hemolysis of human RBC, it also exhibited antimicrobial activity against *E. coli*, *S. aureus*, and *B. cereus*. Docking simulation results revealed that this peptide could bind to the LPS binding site of MD-2, which may prevent LPS from entering the MD-2 binding pocket, and trigger TLR4 signaling pathway. The *Corvus* CATH-2 characterized in this study could aid in the development of novel therapeutics.

## Introduction

The advent of superbugs, which are practically resistant to all of the available antibiotics, prompted a vigorous quest for new non-conventional antimicrobial agents less susceptible to bacterial resistance^1,2^. Antimicrobial peptides (AMP), which have gained a great deal of attention as prospective next-generation antibiotics, are crucial bioactive constituents of the innate immune system that offer host defense and disease resistance in almost all life forms^3–5^. AMPs are rather small molecules, with often less than 100 amino acid residues. The smaller size permits the rapid diffusion and release of AMPs outside the cells which is critical for evoking instant immune response against invading pathogens^5,6^. Most of the naturally existing AMPs display a net positive charge varying from + 2 to + 13 due to the presence of multiple arginine and lysine residues in the peptide sequences. The cationicity of AMPs is crucial for the electrostatic attraction between peptides and the anionic cell membrane of bacteria, leading to the alteration of electrochemical potential of the membrane causing membrane damage and ultimately resulting in bacterial cell death^6–8^. AMPs possess hydrophobic amino acid residues (nearly 50%) and upon interaction with the target membrane they adopt an amphipathic structure. While hydrophobicity is required for the permeabilization of microbial membranes, higher levels can induce loss of antimicrobial selectivity and mammalian cytotoxicity^6,8,9^. These peptides exert antimicrobial activity against various organisms, including bacteria, yeasts, protozoa, enveloped viruses, fungi, mycobacteria, and cancerous cells^10,11^. Because of their non-specific and distinguished approach to microbial killing, AMPs even show efficacy against antibiotic-resistant strains^12,13^. Unlike traditional antibiotics which act on one target, AMPs act on several targets on the cell membrane as well as intracellular targets of the invading bacteria^14^. They display potent antimicrobial efficiency primarily by impairing the membrane integrity of the invading pathogens that cause which leads to cell death^15,16^. Since major targets of AMPs are bacterial membranes, it is more challenging for bacteria to develop resistance to these peptides compared to traditional antibiotics^17^. Though AMP’s primary role is to protect the host from invading pathogens by exerting cytotoxicity, they often also serve as immune modulators in higher organisms. The immunomodulatory activities performed by AMPs includes chemotaxis stimulation, regulation of immune cell differentiation, and initiation of adaptive immunity, all contributing to microbial clearance^7,18^. Important physicochemical properties responsible for biological activity as well as membrane selectivity are charge, hydrophobicity, hydrophobic moment, amphipathicity, and helicity^19,20^. Amphipathic alpha helical AMPs that facilitate the incorporation of the hydrophobic region into the microbial membrane is one of the important classes of membrane-targeting peptides^21^.

AMPs are widely considered to offer several safety advantages over small molecular drugs as the peptide degradation products are amino acids and few peptides accumulate in tissues due to their short half-life^22,23^. Thus peptide therapeutics lessen the risk of metabolite-related complications and health hazards. Many pharmaceutical companies are currently attempting to promote AMP as novel antimicrobial therapeutics^24,25^. Even though these defense peptides have many significant roles; their clinical application has been hampered due to several limitations such as susceptibility to proteolytic degradation, systemic toxicity, high production cost, and poor bioavailability. Modification of natural AMPs has been shown to be an effective strategy to overcome the limitations imposed by nature^26,27^.

Crows are omnivorous scavengers and are considered to be one of the most intelligent animals. *Corvus splendens* (House Crow) is a highly invasive and adaptable avian species in the Corvidae family exhibiting immense ecological flexibility^28,29^. It is a human commensal, living near human settlements and takes the benefit of scavenging opportunity offered by garbage deposits and discarded foodstuffs^29–32^. Given the fact that crows are considered both pests and nuisance by man, crow meat is used as a traditional remedy for stomach ailments, rheumatism, earache, leucoderma, anaemia, malaria and paralysis. Most importantly, they perform valuable service to the ecosystem by removing putrid carcasses or carrions without themselves getting infected^32–36^. Presence of a robust immune system is one of the adaptations for the life of carrion feeders. Their fatal encounters with microbes and toxins play a role that contributes to the development of a robust immune system^37–40^. All organisms possess specialized defence mechanisms tailored for survival in diverse environments. Non-oxidative microbicidal mechanisms, including cationic antimicrobial peptides and proteins, play a major role in providing effective immune response against microbes in birds as avian heterophils are deprived of essential enzymes of oxidative antimicrobial activity^41–43^.

We analyzed and identified several AMPs from the transcriptome of *C. splendens* using high-throughput sequencing technologies (Table 3). No AMPs from this species have been reported. Here we also report the expression profiles of AMP genes. The sequence analysis and characterization of one of the identified AMPs, *Corvus* CATH-2 were also performed in detail.

## Results

### Transcriptome sequencing and assembly

The libraries sequenced using Illumina Nextseq 500 and Illumina HiSeq 2500 platforms generated a total of 23,785,322 and 59,779,517 raw reads, respectively and were submitted to the NCBI-SRA. The summary of the raw reads is shown in Table 1. After quality filtering, 22,961,666 clean reads were obtained from the Set1 sample. Set2 sample produced approximately 53,248,434 (89.07%) clean reads after trimming the adapter sequence and low-quality bases were removed using FilterByTile. The processed reads from both samples were combined before further analysis, yielding 72,098,952 reads with 50% GC content.

**Table 1:** Raw read summary.

Samples	Total sequences	Sequence length	% GC
Set1_R1.fastq.gz	23,785,322	76	47
Set1_R2.fastq.gz	23,785,322	76	47
Set2_R1.fastq.gz	59,779,517	100	51
Set2_R2.fastq.gz	59,779,517	100	51

**Figure 1:**
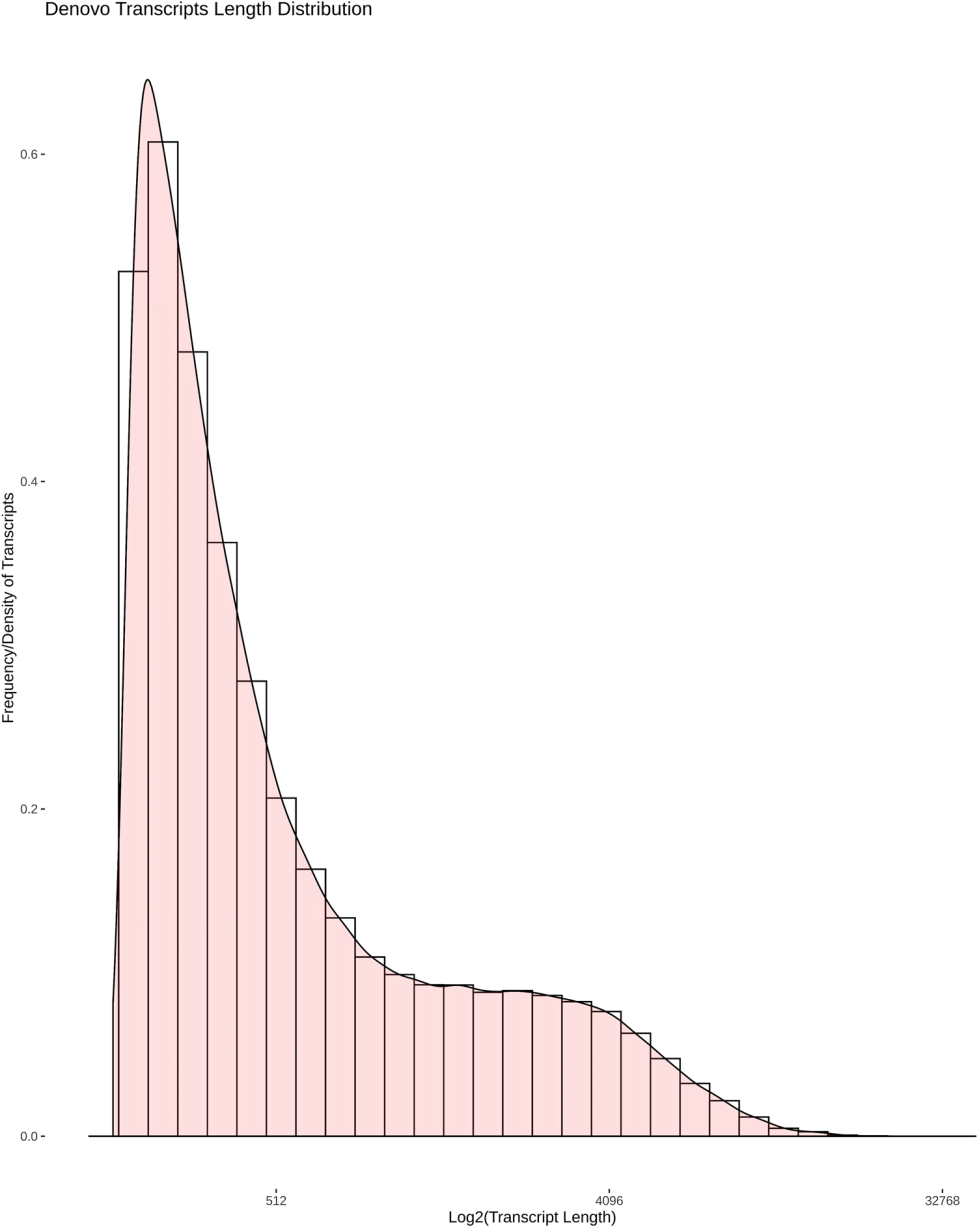
De novo assembled transcript length distribution. Assembled transcript density (Y-axis) was plot against log2 (transcript length) (X-axis).

The assembly of the clean merged reads using the Trinity program generated 3,43,503 transcripts. We analyzed the transcript length distribution and found that the average sequence length was about 1030.40 bp and a N50 length of 2747 bp (Table 2). The length distribution of de novo assembled transcripts is shown in Fig. 1. Trinity assembled transcripts were then clustered using the TGICL tool which produced 74,958 unigenes with a minimum length of 201 bp, the maximum length of 38,887 bp, and N50 size of 3467 bp (Table 2).

**Table 2:** Assembly statistics of transcripts and unigenes.

Assembly statistics	Transcripts	Unigenes
Total sequence length	353,951,778	126,484,523
Minimum sequence length	185	201
Maximum sequence length	38,885	38,887
Average sequence length	1030.40	1687.40
N10	7826	8828
N20	5796	6704
N30	4547	5370
N40	3603	4335
N50	2747	3467
Percent GC	46.62%	46.36%

### Functional annotation

**Figure 2:**
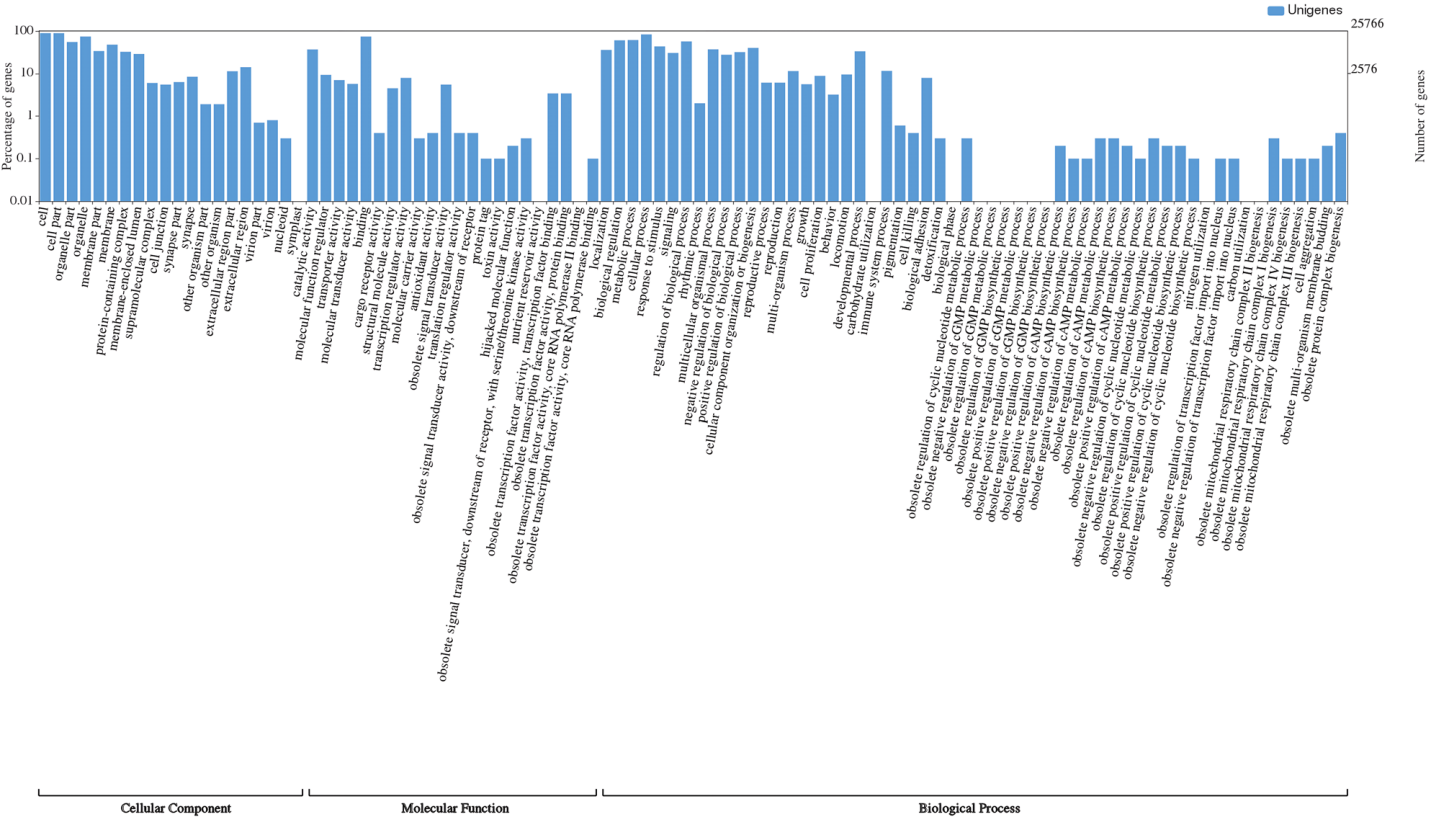
Functional annotation of unigenes based on Gene Ontology.

The assembled unigenes were aligned against SwissProt, Pfam, GO, and KEGG databases using the DIAMOND aligner (e-value 1e−5). In total, 37,559 (50.10%) unigenes were annotated at least in one of the above-mentioned databases (Table S1). Among annotated unigenes, 36,035 (48.07%), 23,518 (31.37%), 25,766 (34.37%), and 35,121 (46.85%) unigenes had homologous sequences in SwissProt, Pfam, GO and KEGG databases respectively. For 25,766 unigenes, we obtained a total of 70,243 GO annotations. These unigenes were categorized into 98 functional groups (Fig. 2), and these groups were classified into three major GO categories, namely cellular component (24,579 unigenes), biological process (23,242 unigenes) and molecular function (22,422 unigenes). The major GO terms for cellular components were cell, organelle and cell part. In molecular function, the greatest number of unigenes was associated with binding and catalytic activity and the dominant subcategories of biological processes were cellular process, biological regulation and metabolic process. KEGG orthology (KO) terms were assigned to a total of 35,121 unigenes and classified into 344 pathways.

### Antimicrobial peptide annotation

**Figure 3:**
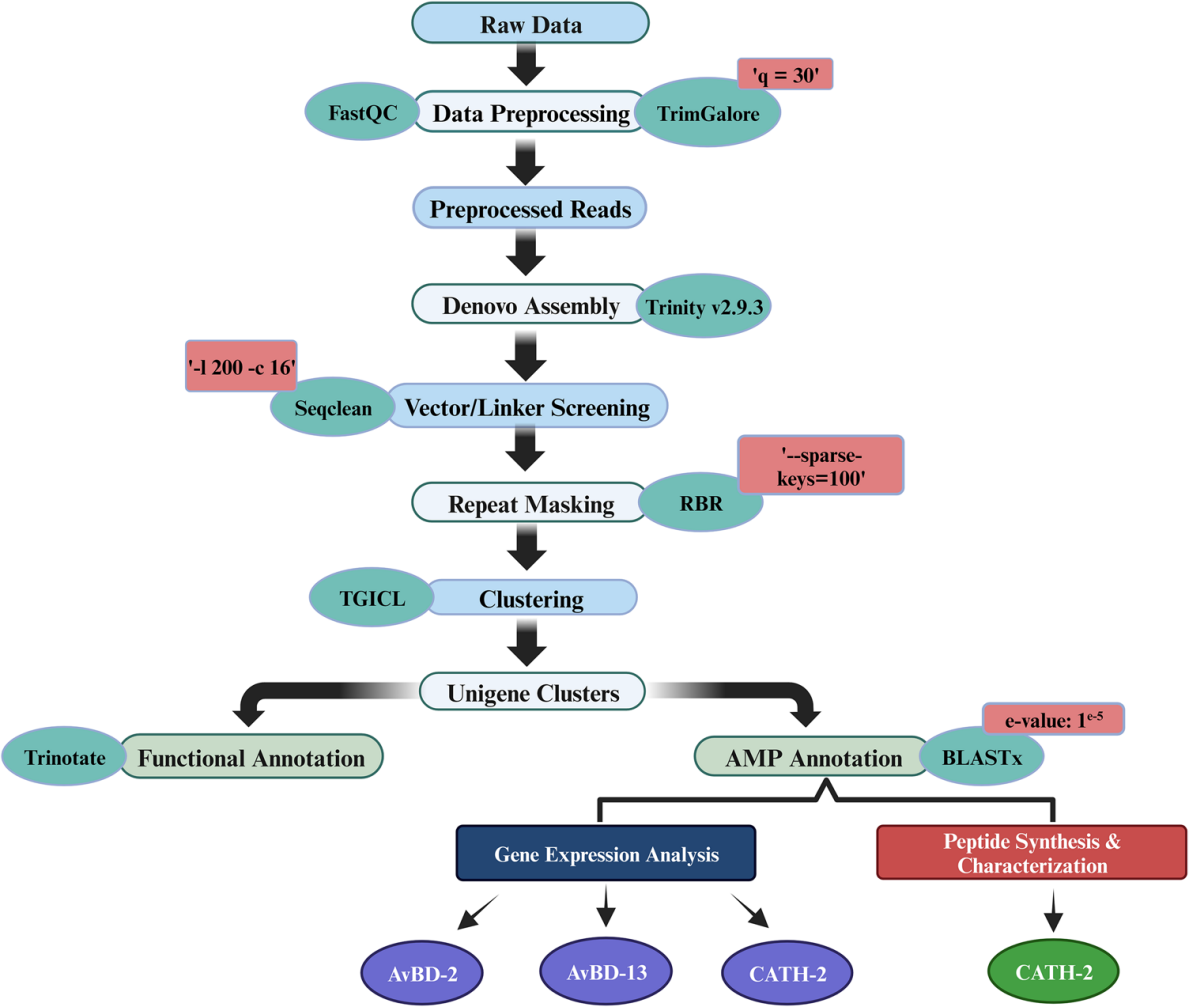
Workflow for transcriptome analysis and AMP annotation.

BLASTx results showed that 578 unigenes were annotated as AMPs in *C. splendens* using antimicrobial peptide databases APD3, CAMP_R3_, and LAMP with an e-value: 1e−5 and similarity score of ≥ 80 (Tables S2, S3 and S4). In this study, we obtained 63 AMPs belonging to more than 20 diverse families and functional classes. Among them, some of the major families were ubiquitin, histones, defensins, cathelicidins, amyloids, LEAP-2, transferrin, neuropeptides, enhancer of rudimentary homolog, hepcidin, and chemokines (Table 3). The workflow for the analysis of transcriptome data and AMP annotation is shown in Fig. 3.

**Table 3:** List of some of the major AMPs identified from *C. splendens* using APD3, CAMP _R3_ and LAMP databases.

Unigenes ID	AMP
CL35Contig1	Ubiquitin
CL10522Contig1	Ubiquicidin
CL516Contig1	Histone H2B 1/2/3/4/6
CL1Contig2038	Histone H4
CL17878Contig1	Histone H2B type 2-E
CL10566Contig1	HNr (histone-derived)
CL10566Contig1	Histone H2A
CL516Contig2	Histone H3
CL63204Contig1	Beta-defensin (Fragment)
CL60147Contig1	Gallinacin-2
CL64604Contig1	Gallinacin-13
CL59522Contig1	Gallinacin-5
CL1Contig2041	Beta-defensin 9
CL1Contig2041	Gallinacin 6
CL26196Contig1	Defensin-like fragment
CL48257Contig1	Beta-amyloid peptide (1–42)
CL60229Contig1	Beta-amyloid peptide (1–40)
CL31589Contig1	Cathelicidin-2
CL8444Contig1	rtCATH-1b (cathelicidin, fish, animals)
CL26031Contig1	Liver-expressed antimicrobial peptide 2
CL26031Contig1	Hepcidin
CL23973Contig1	Neuropeptide Y
CL44565Contig1	Enkelytin
CL44565Contig1	Proenkephalin-A
CL23973Contig1	Bradykinin
CL44349Contig1	Vasoactive intestinal polypeptide
CL52025Contig1	Vasostatin-1
CL44349Contig1	Calcitonin gene-related peptide
CL540Contig1	Enhancer of rudimentary homolog
CL22143Contig1	Ovotransferrin
CL50263Contig1	CCL8
CL7703Contig1	Pleiotrophin-A
CL50263Contig1	CXCL12
CL44565Contig1	Bombinin H7

### Tissue specific expression profiling of AMP genes

Defensins and Cathelicidins are the major group of AMPs found in birds^44,45^. Significant variations in the expression levels of AvBD-2, AvBD-13 and CATH-2 genes were found in different tissues and across crows as per the comparative statistical analysis using multiple *t*-test analysis and one-way ANOVA respectively at P ˂ 0.05 (except two crows in case of AvBD-13 gene and one crow for CATH-2 gene). Difference in expression among the birds may be due to the differences in the surroundings to which they have been exposed.

**Figure 4:**
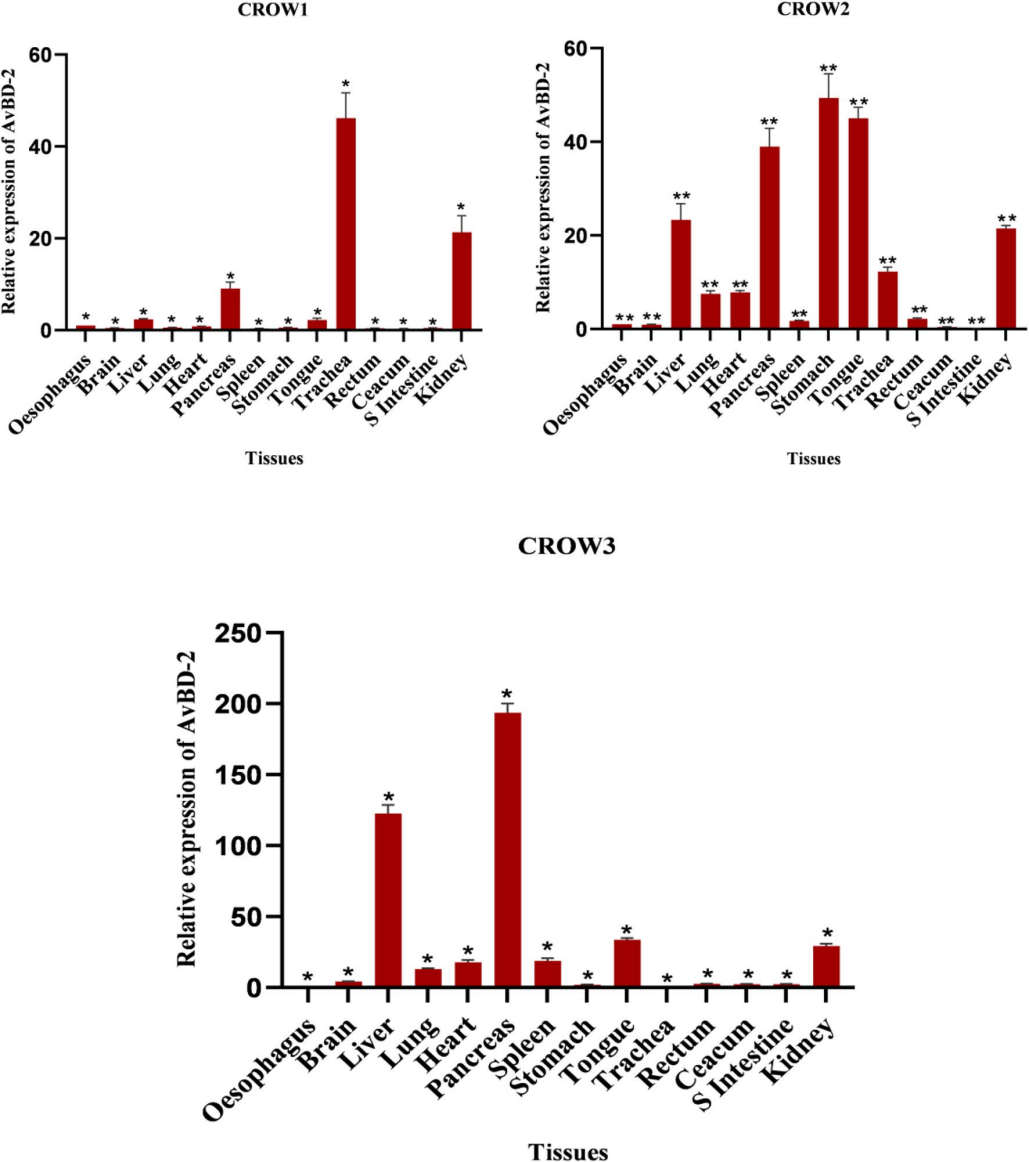
Tissue expression pattern of AvBD-2 gene in three adult *C. splendens.* Asterisks indicate the levels of significant differences *P < 0.05, **P < 0.01, ***P < 0.001.

Defensin secretion represents the first line of defense and their secretions are regulated by the microbial incursions^46,47^. AvBD-2 is an essential element in the innate immune system that aids host defense towards viral pathogens^46,48^. This gene was found to be constitutively expressed at different levels in all the tested tissues (Fig. 4). Among these tissues, the pancreas exhibited higher expression of AvBD-2 mRNA in all the three crows. Higher levels were also detected in the liver, tongue, stomach, and kidney in one of the crows, but moderate expression was observed in other birds. AvBD-2 mRNA was moderately expressed in the heart, brain, rectum, lungs, and spleen. Highly variable expressions were observed in the trachea of all the selected crows. A low to moderate level of expression was observed in the ceacum and small intestine.

**Figure 5:**
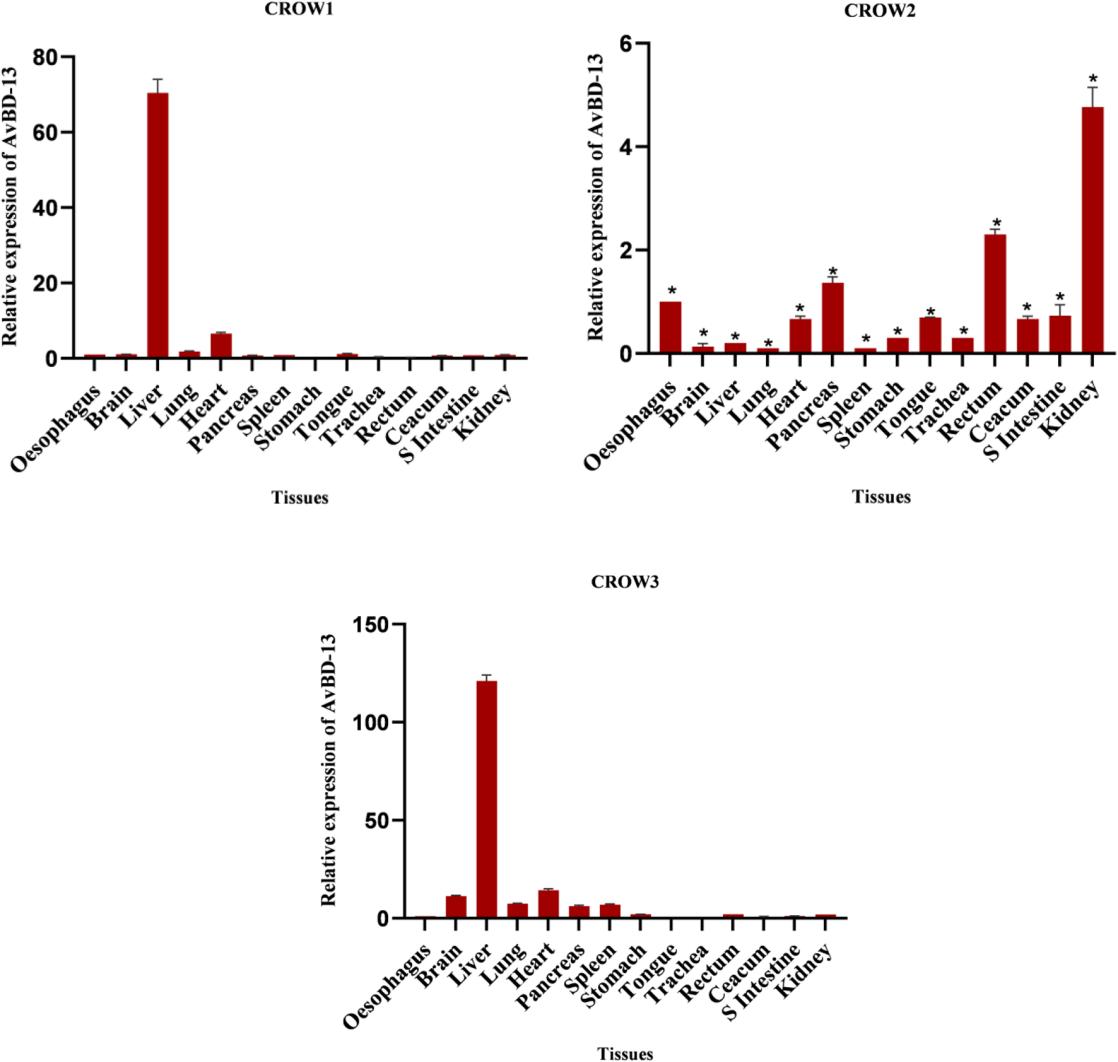
Tissue expression pattern of AvBD-13 gene in three adult *C. splendens.* Asterisks indicate the levels of significant differences *P < 0.05, **P < 0.01, ***P < 0.001. In Crow 1 and Crow 3, P ≥ 0.05 (*NS*)*.*

Except in one crow, the liver exhibited predominant expression of the AvBD-13 gene. This finding is consistent with prior studies that reported a higher level of AvBD-13 mRNA in liver tissue^49^. Moderate to a higher level of expression was detected in tissues like the heart, and kidney, while the rectum exhibited low to high expression. The mRNA was moderately expressed in the tongue, pancreas, stomach, ceacum and small intestine and the rest of the tissues showed moderate to weak expression (Fig. 5).

**Figure 6:**
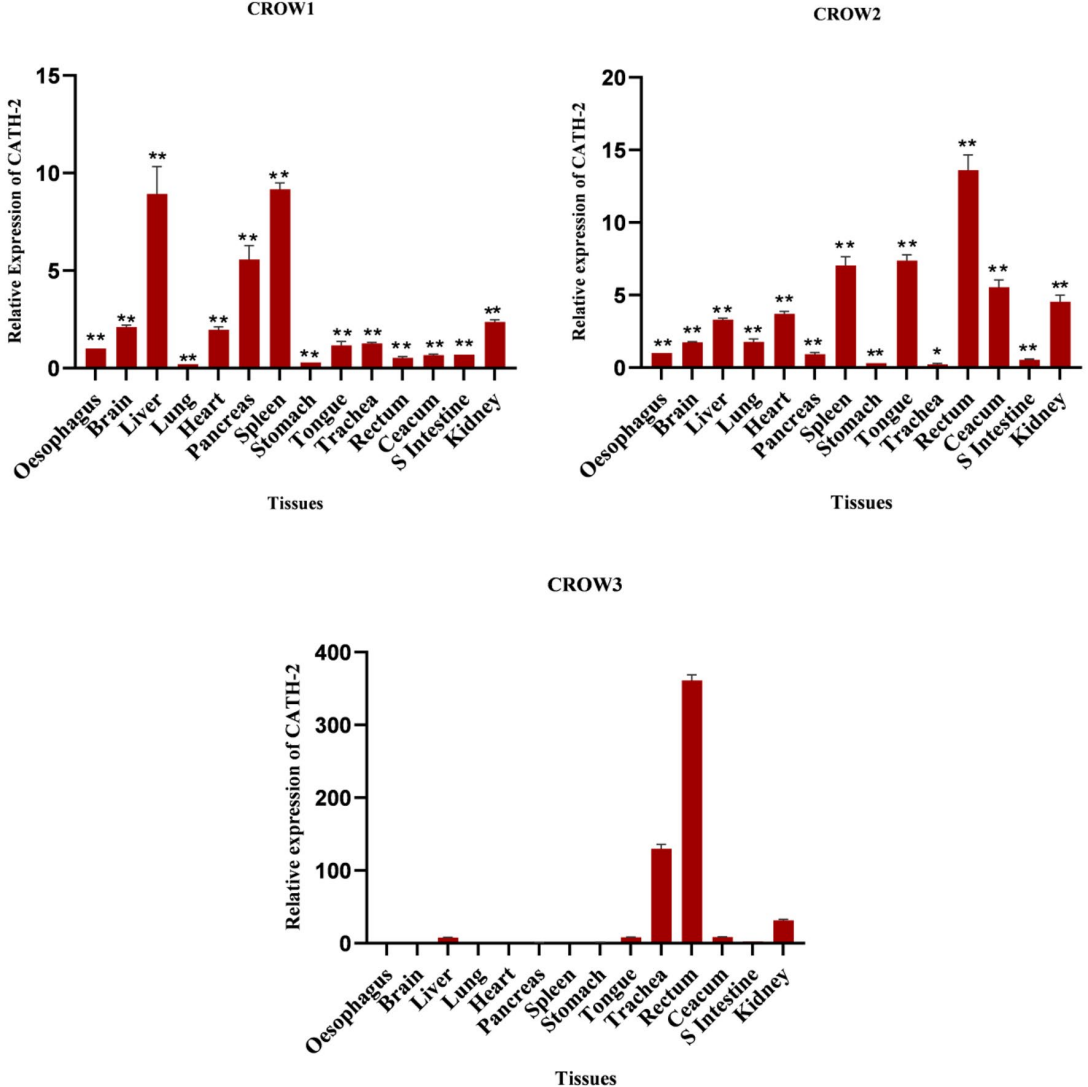
Tissue expression pattern of CATH-2 gene in three adult *C. splendens.* Asterisks indicate the levels of significant differences *P < 0.05, **P < 0.01, ***P < 0.001, In Crow 3 P ≥ 0.05 (*NS*)*.*

An earlier study reported that CATH-1, CATH-2, and CATH-3 are present in a range of tissues, including the gastrointestinal tract, respiratory tract, and lymphoid organs^45,49^. In this study, CATH-2 gene was also found to be constitutively expressed at different levels in all the tested tissues of house crows (Fig. 6). The rectum exhibited higher expression of the gene in two out of three crows, while the liver and tongue showed higher expression in one bird, and moderate expression was observed in other birds. Moderate to high expression was detected in the pancreas. The mRNA was expressed moderately in tissues such as the brain, heart, caecum, and kidney, while the lung, stomach, and small intestine displayed moderate to weak expression. The lowest expression level of this mRNA was found in the stomach. The mRNA showed a varied expression in both the spleen and trachea of the tested crows.

### Sequence analysis and structure modelling of *Corvus* CATH-2 peptide

**Figure 7:**
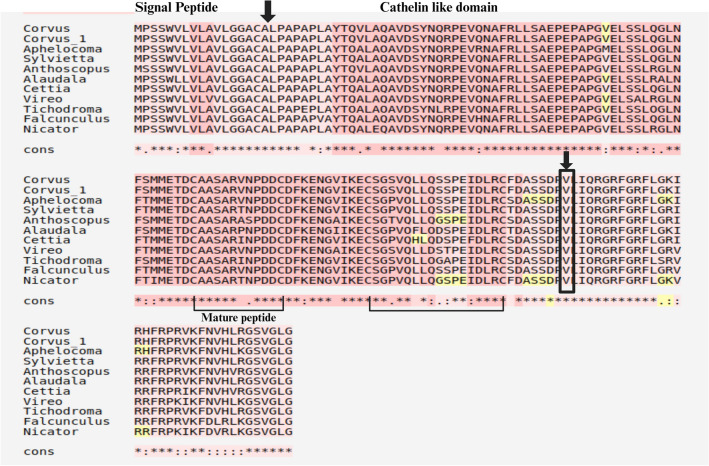
Alignment of amino acid sequences of *Corvus* CATH-2 with other avian CATH-2 homologs. Multiple alignment involved 11 amino acid sequences. Black box indicates Val124. Cystein residues forming intracellular disulphide bonds are connected by solid lines.

The complete nucleotide sequence of CATH-2 included 579 bp long open reading frame encoding a peptide comprising 192 amino acids. The predicted amino acid sequences showed 98.96% similarity with *C. kubaryi.* In *Corvus* CATH-2, a signal sequence of 17 amino acid residues was predicted using SignalP 6.0 software. In sequence alignment, the pro-region of avian CATH-2, which included characteristic cathelin-like domain (CLD) with four conserved cystein residues exhibited a high degree of similarity (Fig. 7). Valine is the optimal cleavage site for the maturation of most cathelicidins^50^. Based on comparison with known CATH-2, Val124 is assumed to be the processing site for elastase in *Corvus* CATH-2, releasing the mature sequence, LIQRGRFGRFLGKIRHFRPRVKFNVHLRGSVGLG.

**Figure 8:**
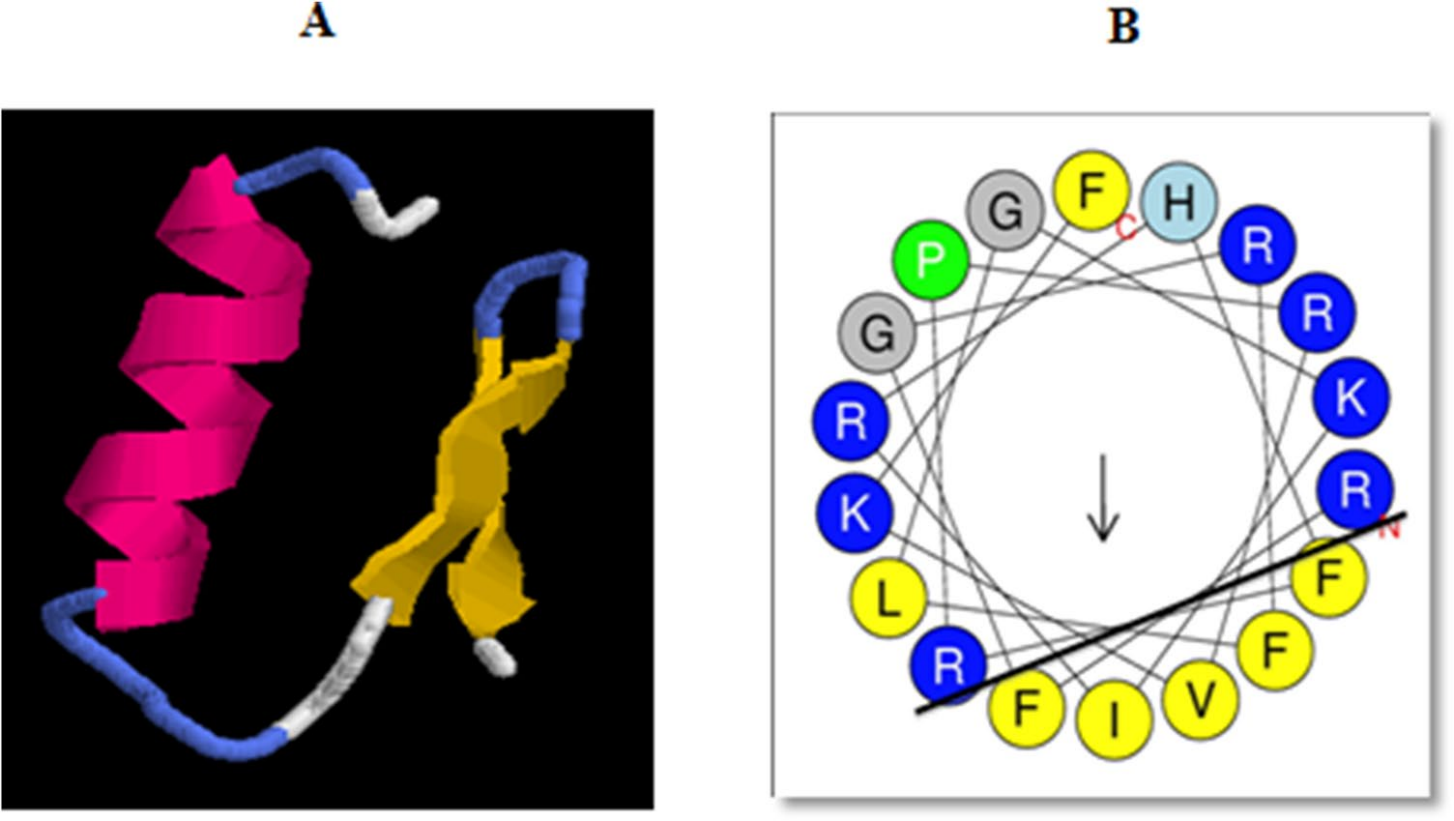
(**A**) Tertiary structure of *Corvus* CATH-2 built by Quark ab initio software. (**B**) Helical wheel plot of *Corvus* CATH-2 in which hydrophilic and hydrophobic residues are separated by a solid line.

The mature peptide possessed a net positive charge (+ 10), 3.97 kDa molecular weight, and a theoretical pI of 13.28. SOPMA results showed the presence of 29.41% alpha helix, 29.41% extended strand, 14.71% beta turn, and 26.47% random coil. The predicted 3D structure of *Corvus* CATH-2 was shown to adopt α-helix and two antiparallel β strands (Fig. 8A). It also included a mild kink caused by a proline residue. Helical wheel analysis showed that 5–22 residues form a distinct amphipathic structure, with the lower side consisting of 5 hydrophobic residues (FFVIF) and the upper side possessing mainly hydrophilic residues (Fig. 8B).

### MD simulations

**Figure 9:**
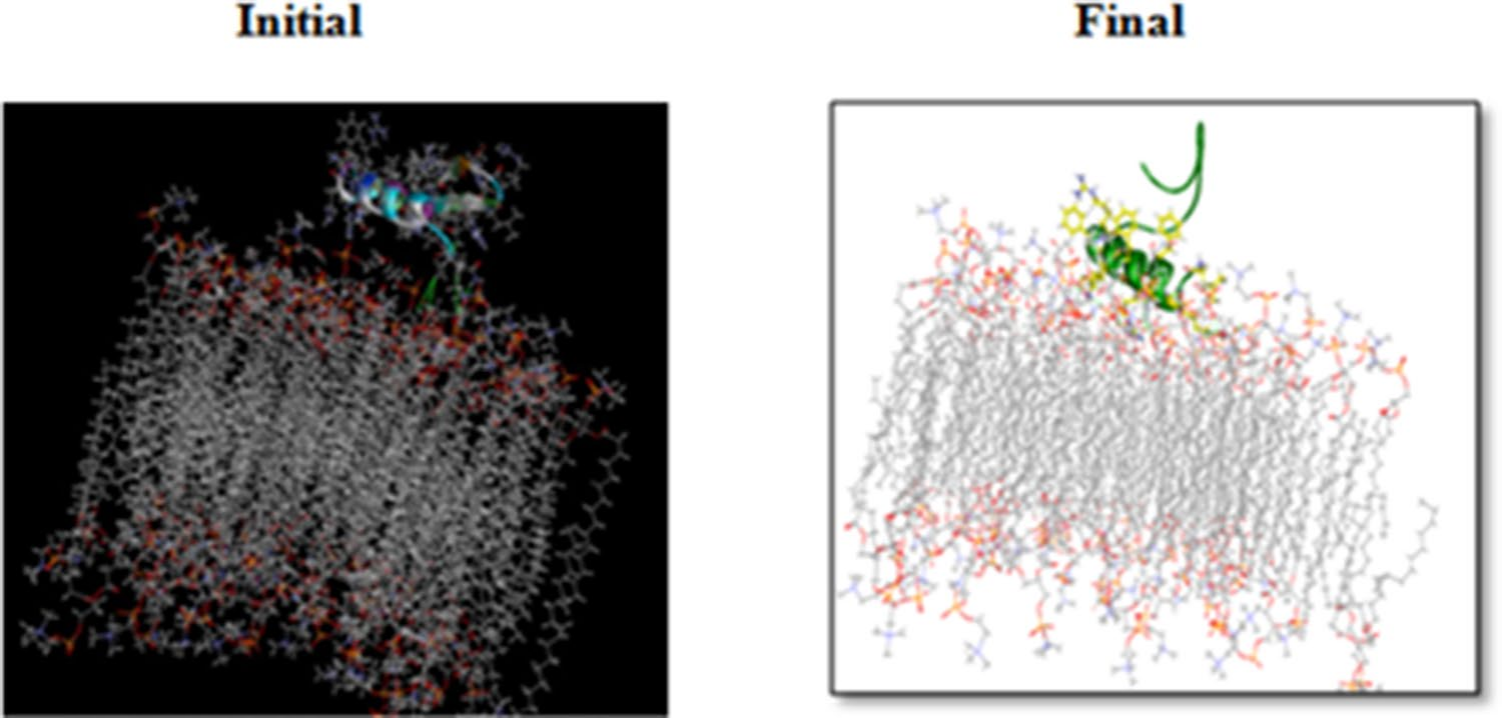
Representative structures of *Corvus* CATH-2/mixed membrane interaction.

**Figure 10:**
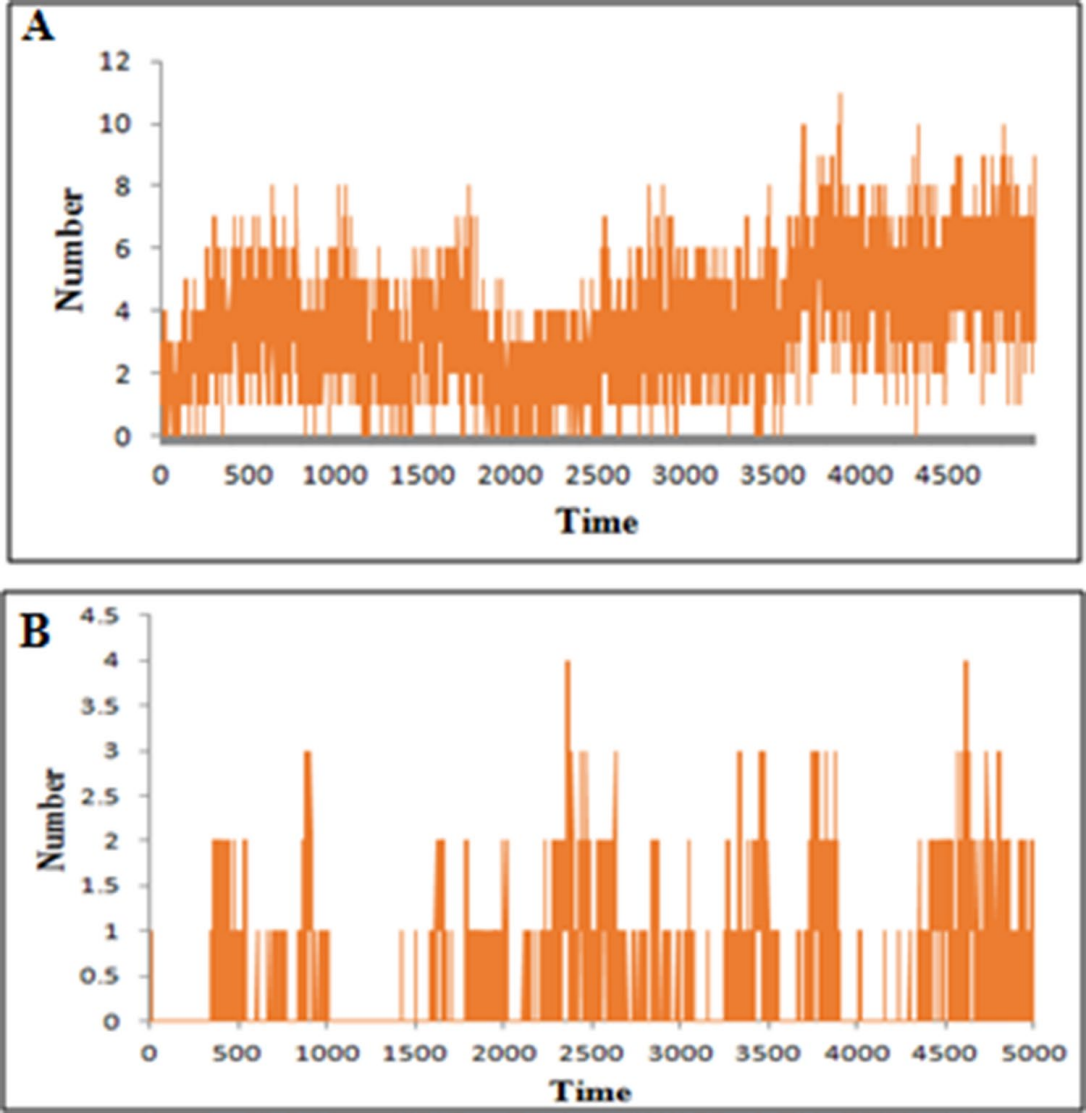
Number of hydrogen bonds formed between (**A**) *Corvus* CATH-2 and mixed membrane, (**B**) *Corvus* CATH-2 and POPC membrane.

To understand the mechanism of microbial killing and cell selectivity of the peptide, we performed a 10 ns MD simulation of the peptide with two model membranes, mixed membrane and POPC alone. The basic principle for antimicrobial activity appears to be the electrostatic interaction of peptides with anionic molecules on the membrane^5^. The hydrophilic side of the amphipathic helix of CATH-2 was shown to adhere to the mixed membrane during simulation (Fig. 9). Certain hydrophobic residues were also observed to be involved in the membrane interaction. Hydrophobicity of the peptide is crucial for membrane penetration, which is necessary for its effective functioning^20^. At the end of the simulation, the number of hydrogen bonds between peptide and mixed membrane increased (Fig. 10A).

**Figure 11:**
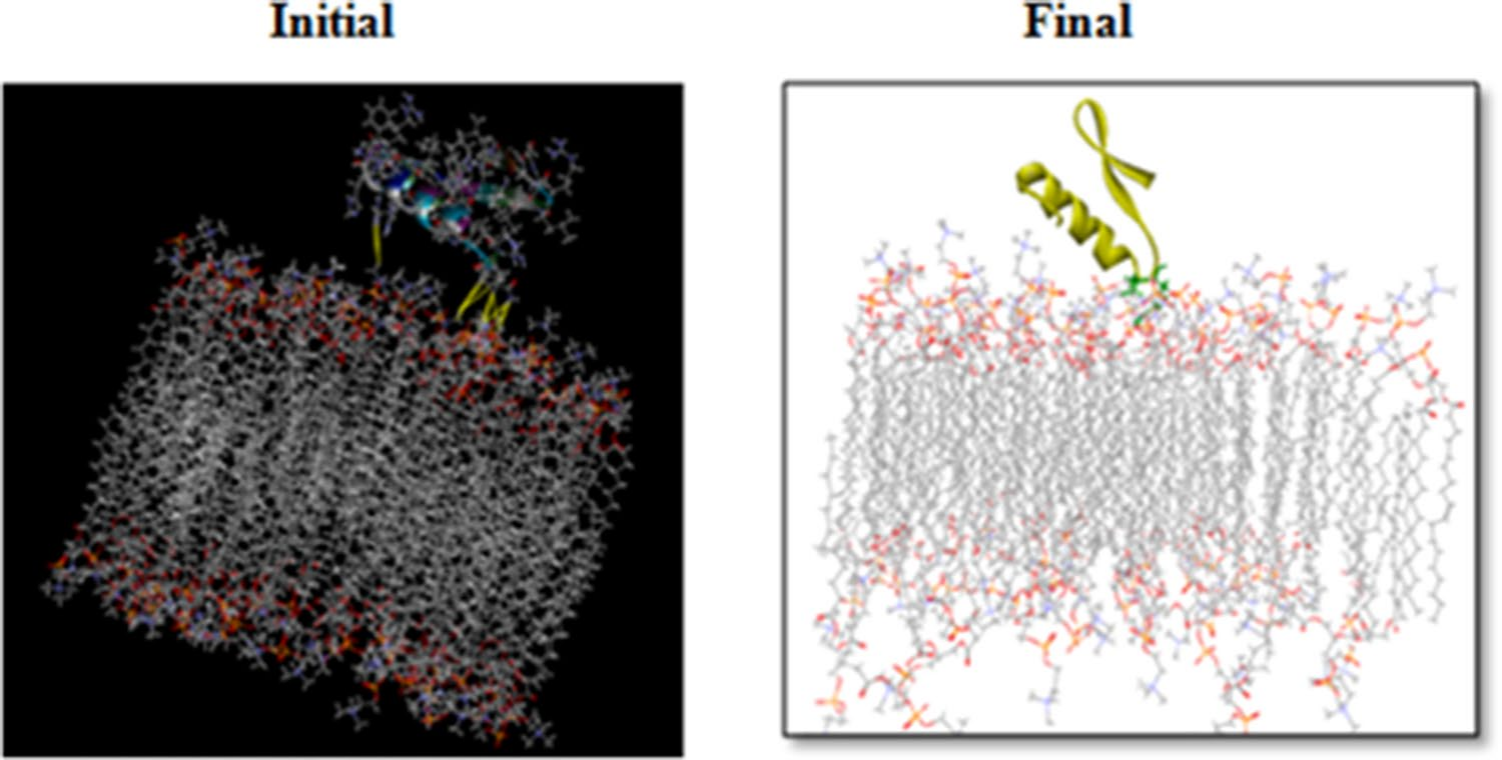
Representative structures of *Corvus* CATH-2/POPC membrane.

The Leu1 and Ile2 at the N-terminus and His16, Pro19, and Arg20 at the kink region of CATH-2 were found to mediate its interaction with the POPC membrane. Towards the end of the simulation, the kink region was drawn closer to the membrane (Fig. 11). These five residues were found to be involved in van der Waals interactions, while Arg20, the cationic residue at the kink region, formed hydrogen bonds as well. The number of hydrogen bond interactions between the peptide and POPC membrane during the entire simulation is depicted in Fig. 10B. In comparison to the mixed membrane, the peptide formed fewer hydrogen bonds with the POPC membrane.

### Structural characterization

**Figure 12:**
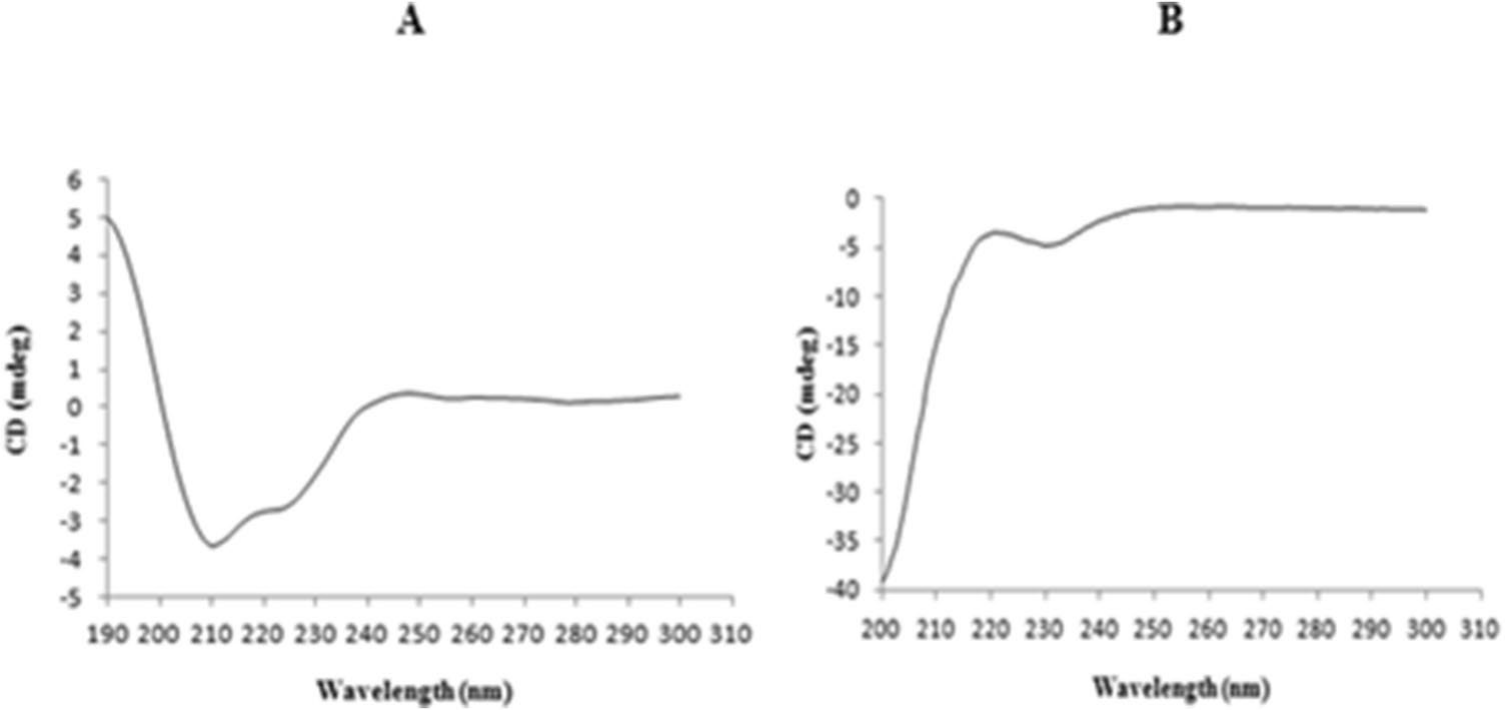
Circular dichroism spectra of *Corvus* CATH-2 in (**A**) 50% (TFE)/water and (**B**) water. The mean ellipticity of residues was plotted against the wavelength.

**Figure 13:**
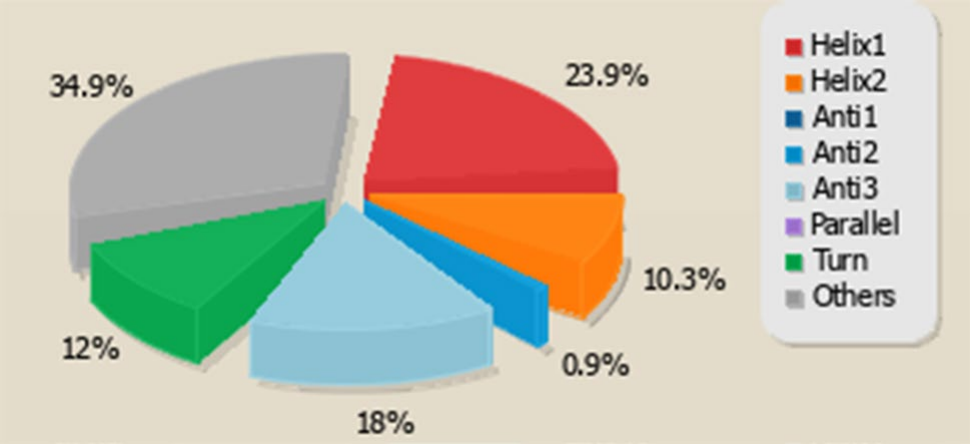
Secondary structures prediction of *Corvus* CATH-2 using BeStSel analysis server.

CD spectrum of the synthesized peptide in membrane mimicking medium of 50 percent v/v TFE/H_2_O which mimics the hydrophobic environment of the microbial membrane^51^ revealed a single positive peak at 190 nm and 2 negative peaks at 208 nm and 222 nm, respectively, indicating an alpha-helical conformation. However, the peptide displayed an unstructured or random coil conformation in water (Fig. 12A,B). The secondary structures estimated from the obtained CD spectrum using BeStSel reported the existence of 34.2% helical fraction, which included 23.9% of regular helix and 10.3% of distorted helix (Fig. 13).

### Antimicrobial activity

**Figure 14:**
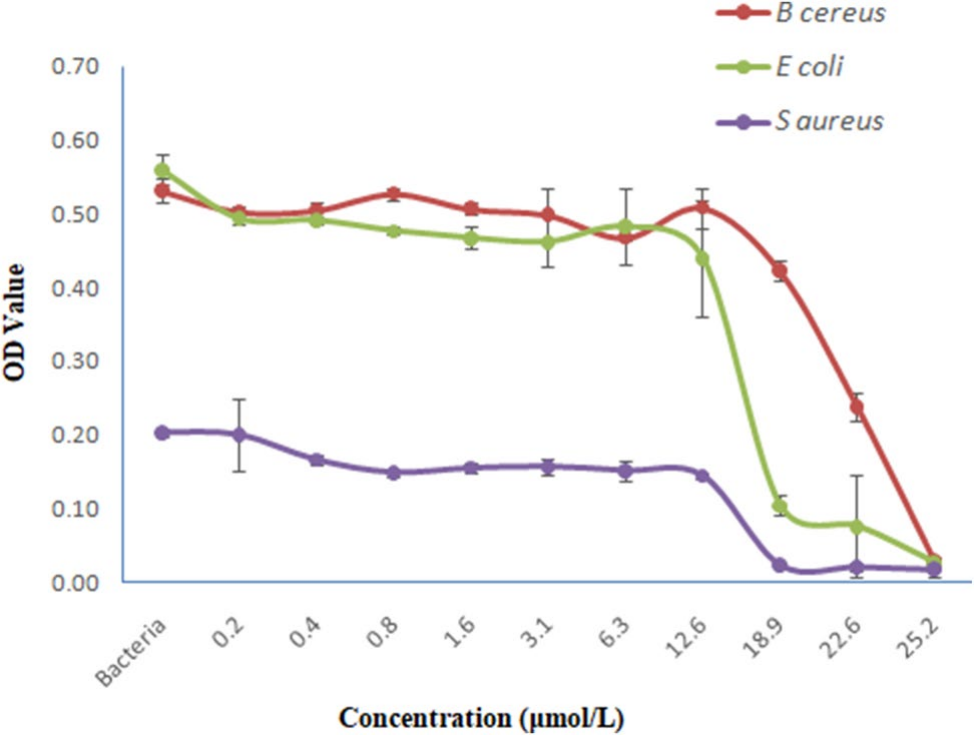
Antimicrobial activity of *Corvus* CATH-2 against *B. cereus*, *E. coli* and *S. aureus*. Data shown are mean ± standard deviation from three independent experiments. Inoculum (respective strains)—positive control.

The MIC of *Corvus* CATH-2 was determined for three strains of bacteria such as *S. aureus*, *E. coli*, and *B. cereus*. Although the tested organisms were susceptible to *Corvus* CATH-2, MIC values were found to be 18.87 μM for *S. aureus*, 22.64 μM for *E. coli* and 25.16 μM for *B. cereus* (Fig. 14)*.*

### Hemolytic activity

**Figure 15:**
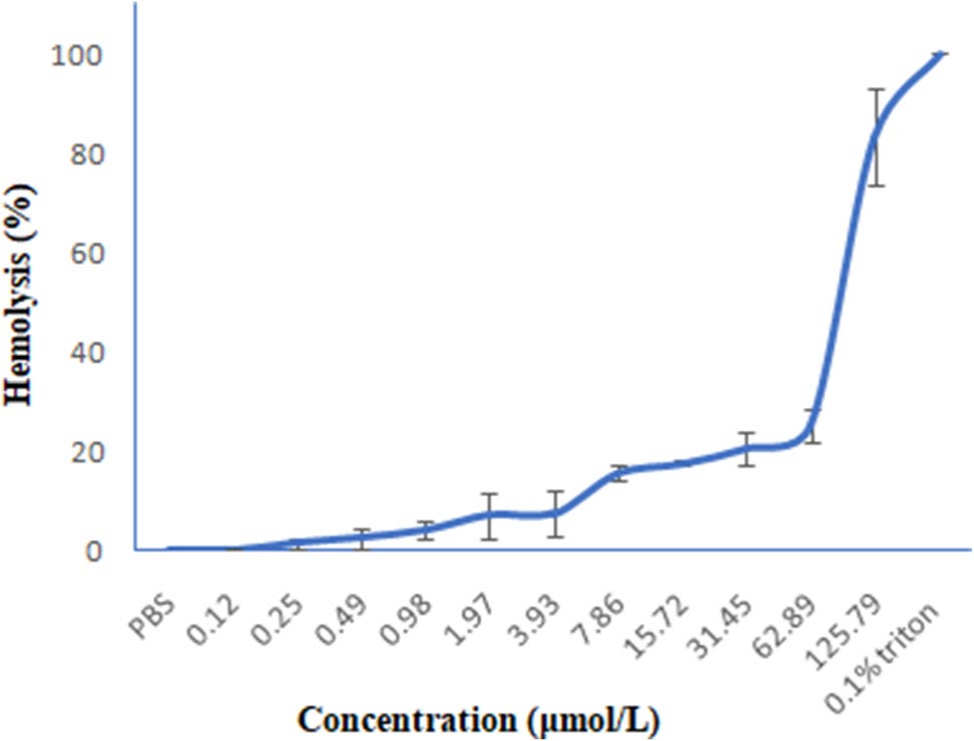
Hemolytic activity of *Corvus* CATH-2 against human erythrocytes. Data shown are mean ± standard deviation from three independent experiments. 0.1% Triton—positive control, PBS—negative control.

The hemolytic activity of AMPs against human RBC is generally considered as an indication of their toxicity towards mammalian cells^52,53^. The synthesized peptide induced 22% hemolysis at a concentration up to 62.89 µM (Fig. 15).

### Interaction of TLR4 and *Corvus* CATH-2

**Figure 16:**
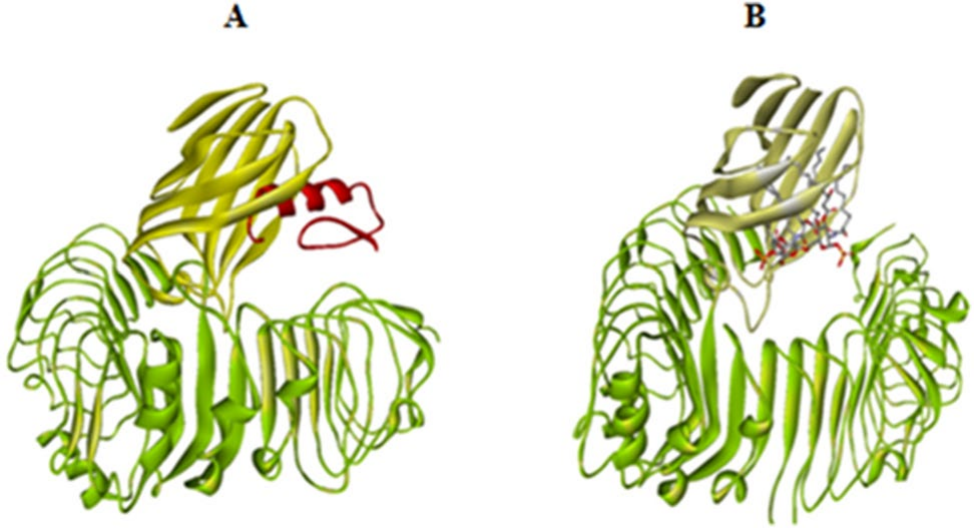
Structure of (**A**) TLR4–MD-2–*Corvus* CATH-2 complex, TLR4, beta sheeted MD2 cup and CATH-2 are displayed in green, yellow and red respectively. (**B**) TLR4–MD-2–LPS complex, TLR4 and beta sheeted MD2 cup are colored in green and yellow respectively, LPS is shown in grey and red sticks.

The interaction of LPS, a pathogen-associated molecular pattern (PAMP) with pattern-recognition receptors (PRR) like TLRs, activates innate immunity. TLR4 alone is incapable of detecting LPS, necessitating its association with MD2 to form TLR4–MD2 complex in which MD2 interacts with LPS. The binding of LPS causes dimerization of the TLR4/MD-2 complex, resulting in the activation of several signalling pathways. According to previous studies cathelicidins can neutralize LPS, and TLR4 is a binding target for these peptides^54,55^. The binding mechanism of *Corvus* CATH-2 to TLR4–MD-2 complex was studied by docking simulation. The docked structure (TLR4–MD-2–*Corvus* CATH-2 complex) was compared to the crystal structure of mouse TLR4–MD-2–LPS complex (PDB ID: 3VQ2) obtained from PDB (Fig. 16A,B). The docking result showed that CATH-2 was well positioned in the LPS binding pocket in MD-2 which implies that the peptide can bind to the TLR4/MD-2 complex. Several MD-2 residues including Ile52, Leu54, Val61, Leu78, Ile80, Leu87, Arg90, Glu92, Leu94, Tyr102, Ile117, Pro118, Phe119, Ser120, Phe121, glu122, Gly123, Ile124, Phe126 and Tyr131 were found to be involved in the binding of both CATH-2 and LPS to TLR4–MD-2 complex. This interaction may prevent LPS from binding to MD-2 and activating the signaling pathways.

## Discussion

The alarming evolution of multidrug-resistant superbugs has motivated the discovery of novel antimicrobial agents. Antimicrobial peptides have been proposed as prospective therapeutic candidates due to their broad activity spectrum, immunomodulatory effects, and the reduced possibility of inducing microbial resistance. These defense peptides are ubiquitously present as a part of the host defense systems from invertebrates to mammals, indicating their immunological relevance^3,54,55^. The scavenging habit, heightened immunity, flexible diets, and adaptability of the house crow make it an ideal candidate for studying AMPs.

As AMPs showed a tissue-specific expression pattern in chicken^56,57^, pooling tissues from multiple organs was perceived as more relevant than examining individual tissue types. In this study, we were able to annotate 37,559 unigenes. The inadequate number of sequences from closely related passeriform avian species in public databases accounts for low annotation value^58^. A total of 25,766 unigenes were assigned in GO annotations and were divided into 98 subcategories of three major functional ontologies, including biological process, cellular component, and molecular function. Furthermore, we identified 35,121 unigenes mapped to 344 pathways. In *Corvus macrorhynchos* also more than 300 KEGG pathways were identified^58^, which is close to our finding.

Defensins, cathelicidins, and LEAP-2 are the major families of avian AMPs that have been reported to exhibit a broad spectrum of antimicrobial activities^59–61^. Hepcidin, formerly known as LEAP-1, not only acts directly against pathogens but also as a central regulator of iron homeostasis, which is impaired by inflammation or infection. Transferrin is also related to iron homeostasis^62–64^. Neuropeptide Y, enkelytin, bradykinin, vasoactive intestinal polypeptide, calcitonin gene-related peptide, vasostatin-1, and proenkephalin-A were among the neuropeptides obtained in this study. Neuropeptides and AMPs share several structural and physicochemical properties include low molecular mass, cationicity, and amphipathicity. These characteristics allow neuropeptides to interact with the anionic constituents of the microbial cell envelope, resulting in membrane disruption, and microbial cell lysis^65^. A previous study revealed the antimicrobial efficacy of neuropeptide Y, calcitonin gene-related peptide and vasoactive intestinal polypeptide against *Escherichia coli*, *Pseudomonas aeruginosa* and *Candida albicans*^66^. Histones, a highly conserved and universal component of the innate immune system, can kill microbes directly and induce inflammatory reactions^67^. The antimicrobial property of amyloid β-protein (Aβ) was reported against eight clinically important microbes and showed striking resemblance to the human AMP, LL-37^68^.

Given the importance of AMPs in innate immunity and the unavailability of data on the tissue specificity of AMPs at the mRNA level in *Corvus* species, we examined the expression pattern of 3 AMPs in different tissues of *C. splendens*. In the present study, the pancreas crows exhibited the robust expression of AvBD-2 followed by the liver, tongue, stomach, and kidney. The production of pancreatic AMPs is crucial in the context of diabetes and pancreatitis. The metabolic regulation of pancreatic AMP expression is particularly intriguing in case of diabetes, where upregulation of defensin expression from elevated blood glucose level may be a protective factor in the early phase of disease^69,70^. In chicken, Van Dijk (2007) reported the presence of AvBD-2 mRNA in the lung and spleen^71^. Consistent with this finding, the current study also showed moderate expression of this gene in the lung and spleen. A higher level of AvBD transcripts in the kidney could be attributed to their role in the development of an efficient antimicrobial barrier to the urinary tract from microbial infection.

The current analysis showed that the liver showed a strong expression of AvBD-13 followed by the heart and kidney. The expression of the examined *Corvus* AvBD mRNA in the tongue may reflect their role in maintaining the level of commensal as well as being effective against harmful microbes in the oral cavity. The first AMP detected in the oral epithelium was β-defensin, identified in the bovine tongue^72^. Like chicken β-defensins, low to medium expression of AvBDs was detected in the small and large intestines of crows. The fermentation of poorly digestible substances takes place in the caecal pouches, which get emptied only once every 8 h on average. So an efficient local immune barrier can be expected at this site to prevent infection. In addition, it has recently become apparent that intestinal AMPs not only ward off ingested pathogenic microbes but also regulate and shape the composition of the gut microbiome^71,73,74^.

In chicken, CATH-1–3 and* -*B1 are widely expressed throughout the digestive tract, genitourinary tract, respiratory tract and lymphoid organs^45,56,75^. In our study also, we found that *Corvus* CATH-2 displayed distinct tissue-specific expressions. The lowest level of CATH-2 mRNA expression was detected in the stomach. Its expression in immunologically important tissue such as the spleen implies that these peptides may be involved in the maturation and development of adaptive immunity. Their distribution in several non-immune tissue types may be suggestive of their function beyond host protection^56^. According to Chromek, cathelicidins display antimicrobial activity against most common uropathogens^76^.

Cathelicidins represent a major group of host defence peptides prevalent in almost all vertebrate species that play critical roles in providing innate immunity. They are linear peptides that mostly adopt amphipathic α-helix structure, which is the most successful conformation that allows the efficient interaction with lipid bilayer^21,77^. These AMPs exhibit both antimicrobial and immunomodulatory functions^78–80^.

Recognizing the selectivity strategies by which host defense peptides interact with mammalian or bacterial membranes would enable us to modulate their selectivity to improve antimicrobial efficacy while reducing cytotoxicity^81^. Several small cationic peptides adopt amphipathic α-helical conformation and are believed to be essential for destroying the membrane integrity^82^. Based on the simulation study using *Corvus* CATH-2 and mixed membrane, we found that the hydrophilic region of the amphipathic helix of the peptide is responsible for initial binding to the membrane. Thus the cationic residues in the amphipathic helix seem to have a significant role in microbial killing. The first contact point between peptide and the membrane is perhaps the spot of antimicrobial activity since they impair the membrane function to induce microbial killing^83^. However, CATH-2 showed an inclination towards the neutral membrane, hence may be hemolytic. The critical importance of the central kink region in the function of cell toxicity was reported in chicken fowlicidin-3^84^. A highly hemolytic scorpion venom AMP, pandinin 2 (Pin2), also has a central proline kink and is related to its ability to generate pores in human erythrocytes^85^. In concordance with the previous reports, we also found that kink region residues, including His16, Pro19, and Arg20 were primarily responsible for the *Corvus* CATH-2-POPC membrane interaction. Thus, MD simulation study sheds light on the initial stages of the action mechanism of CATH-2 of *C. splendens.*

The secondary structure of the CATH-2 peptide was analyzed using a computational approach as well as circular dichroism spectroscopy. As predicted by computational analysis, the CD spectrum of the synthesized peptide exhibited a typical α-helical structure in the membrane environment. The peptide adopts an amphipathic α-helical structure, with polar and non-polar amino acids on the opposite side of the helix, as is typical of linear cationic AMPs^55^. Previous studies have reported that the transition from unstructured conformations in the aqueous media to the amphipathic α-helix in the membrane environment is crucial in peptide partitioning to the membrane and is correlated with antimicrobial efficacy^51,86,87^.

We further examined the antimicrobial efficacy of the synthesized peptide using a modified broth microdilution method. The peptide exerted antimicrobial activity against *E. Coli*, *B. cereus*, and *S. aureus* with MIC greater than 12.58 μM. The MIC values of chicken fowlicidins, quail CATH-2, duck CATH and pigeon Cl-CATH-2 are in the range 0.4–2.5 μM, 0.3–2.5 μM, 2.0–4.0 μM and 2.27–9.08 μM respectively^50,88–90^. The differential potency of AMPs could be explained by the structural variations in their alpha-helical content and amphipathic balance. In pigeon, Cl-CATH-2 exerted lower antimicrobial activity than Cl-CATH-3, which possesses higher alpha-helical content and amphipathicity compared to Cl-CATH-2^90^. Reduced alpha-helical content and hydrophobicity of *Corvus* CATH-2 which is nearly similar to pigeon CATH-2 may underlie its lower activity. Yu et al. also reported that the presence of structurally neighbouring cationic amino acids, including arginine and lysine on the outer side of Cl-CATH-2 peptide aided its interaction with the anionic cell surface and caused membrane disruption^90^. In accordance with this, in our MD simulation analysis, we found that the cationic residues on one side of the amphipathic helix of *Corvus* CATH-2 were observed to interact with the anionic mixed membrane.

The most prevalent side effect of cathelicidins is cytotoxicity to mammalian cells^50,91^. Fowlicidin-2 showed 50% lysis of human and chicken erythrocytes at 15–20 μM and dCATH caused 50% hRBC lysis at 20 μM^50,89^. *Corvus* CATH-2 displayed a lower hemolytic activity in comparison with fowlicidin-2 and duck cathelicidin (dCATH) and caused 22% lysis upto 62.9 μM. Because of its lower haemolytic activity, *Corvus* CATH-2 could be optimized to improve its properties in order to use it as an efficient therapeutic agent. The development and optimization of derived peptides based on natural AMPs have recently become research focus^92^. Several studies have shown that modifying natural AMPs can augment their antibacterial action^93,94^. For example, the replacement of phenylalanine with a halogenated phenylalanine analog in jelleine-1, a small AMP isolated from the royal jelly of *Apis mellifera* enhances its antibacterial efficiency invitro. By halogenations, the stability of jelleine-1 is enhanced 10–100 times^95^. The cyclisation of the backbone of arenicin-1 resulted in increased antimicrobial efficiency and ability to combat drug resistant strains, but had no impact on cytotoxicity^96^. C-terminal amidation of maximin H5 enhances antimicrobial activity without increasing cytotoxicity^97,98^. The findings of hemolytic activity assay confirmed our MD simulation study wherein CATH-2 peptide showed an inclination towards the neutral POPC membrane and the residues of the kink region were found to be primarily responsible for peptide neutral membrane interaction.

In addition to a wide range of antimicrobial activities, many cathelicidins have the ability to directly bind and neutralize lipoteichoic acid (LTA) and lipopolysaccharide (LPS) and suppress septic shock caused by bacteria^80,99–101^. Earlier studies have shown that cathelicidin-PY identified from frog skin inhibits LPS-induced TLR4 expression^101^. Another report showed that chicken CATH-2 causes a non-immunogenic mode of destruction of *P. aeruginosa* as well as restricts inflammation by preventing TLR4 activation^102^. TLR4 is one of the binding sites of sea snake cathelicidin, Hc-CATH and this defense peptide performs its anti-inflammatory activity by inhibiting the TLR4 driven inflammatory reactions triggered by LPS. Molecular docking studies in sea snake and pigeon demonstrated that apart from direct LPS neutralization, the interaction of Hc-CATH (sea snake) and Cl-CATH-2 (pigeon) with the TLR4/MD-2 complex spans the LPS-binding region on MD2, thus preventing LPS from binding to MD-2^55,90^. In the current study, molecular docking results also showed the interaction of *Corvus* CATH-2 with the LPS binding site of MD-2 without directly binding to TLR4. This interaction may hinder the entry of LPS into the MD-2 pocket and initiate the TLR4 signalling pathway.

## Conclusion

This study reports the transcriptome analysis of *C. splendens* as well as valuable genetic resources for the identification of genes associated with the immune system and other molecular pathways. Several antimicrobial peptides and proteins belonging to more than 20 different families were identified. The enormous dataset of these defense peptides identified from this study offer an attractive resource for further molecular analysis as well as target for the development of novel biotherapeutics. The use of gene editing tools and technologies for manipulating AMP coding genes offers immense scope for future research and investigations. In addition, computer-aided AMP discovery can be exploited for the screening of new peptides with pharmaceutical potential.

The expression level of AMPs if precisely mapped may have clinical significance. A significant difference in the degree of AMP expression may serve as biomarkers for various disorders and also used for the detection and diagnosis of microbial infections. In addition, the information on the tissue specificity of these defense peptides may be helpful in the development of drugs specific to different organs/organ systems.

CATH-2 peptide showed antibacterial activity and a low hemolytic activity. Docking simulation and analysis of *Corvus* CATH-2 and TLR4 indicates probable immunomodulatory role of this peptide. All these attributes of *Corvus* CATH-2 encourage further research to develop the peptide into a potential anti-infective drug with enhanced therapeutic profiles. Rationally designed novel analogs of AMPs possessing ideal pharmacokinetic properties can be used to save humans and animals from various infectious diseases that are not treatable with conventional antibiotics.

## Materials and methods

### Ethics statement

Permissions for conducting the study were granted by the following agencies, (i) Principal Chief Conservator of Forests (Wildlife) and Wildlife Warden, Kerala, India (ii) Institutional Animal Ethics Committee (IAEC), College of Veterinary and Animal Sciences, Pookode, Kerala, India (Approval number: IAEC/COVAS/PKD/I7/20I9) and (iii) Institutional Human Ethics Committee (IHEC) of Central University of Kerala, India (Approval number: CUK/IHEC/2019/060).

### Tissue sampling and RNA isolation

Fourteen tissues, including tongue, oesophagus, stomach, liver, spleen, pancreas, small intestine, caecum, rectum, trachea, lung, heart, brain and kidney, were obtained from three healthy adult *C. splendens* for this study. The crows were euthanized intraperitonially with pentobarbitone sodium. Its tissues were immediately dissected and stored at − 80 °C in RNALater^®^ solution (Ambion^®^) until use.

In this study, two sets of transcriptomes from 14 tissues of three crows were pooled and sequenced. For Set1, the total RNA extracted from a pooled sample of 14 tissues from a single crow was used, whereas Set2 comprises of RNA extracted from the pooled tissues of two crows. Total RNA was separately extracted using Trizol RNA isolation protocol^103^. The purity and concentration of the extracted RNA were measured using Qubit as well as Nanodrop. The integrity of RNA samples were assessed using Agilent 2100 RNA Bioanalyzer (Agilent Technologies) and Agilent 2200 Tape station system (Agilent Technologies), respectively. High quality RNA samples with OD230/260 and OD260/280 values ≥ 1.8 and RNA integrity (RIN) ≥ 6.5 were used in cDNA library construction and sequencing.

### cDNA library construction and sequencing

For Set1, RNA-Seq library construction, as well as sequencing was done at the sequencing facility of Genotypic technology Pvt. Ltd., Bangalore, India. Sequencing library was prepared using Illumina TruSeq RNA library protocol described in the TruSeq RNA Sample Preparation Guide (Part # 15026495 Rev. F). The quantification and qualification of the library were assessed using Qubit and High Sensitivity Bioanalyzer Chip (Agilent), respectively. Paired-end sequencing was done on an Illumina NextSeq500 platform. The obtained raw reads were submitted to National Center for Biotechnology Information (NCBI) with Short Read Archive (SRA accession number: SRR10541574) under the bioproject PRJNA591600.

The library preparation and transcriptome sequencing for the Set2 were carried out at Scigenom lab private limited, Kochi, Kerala. Total RNA extracted from two different crows was pooled together prior to library construction. RNA-Seq library was generated according to the Illumina TruSeq RNA Sample Preparation v2 Guide (Part # 15026495 Rev. F). The quantity and quality of the prepared library were determined using the Qubit and Agilent 2200 Tapestation systems, respectively. The library was then sequenced on an Illumina HiSeq2500 platform. The raw reads were deposited at the NCBI SRA, as SRR10560921 under PRJNA591600 bioproject.

### Processing of raw data

Raw sequencing reads (SRA: SRR10541574 and SRR10560921) were subjected to quality check using FastQC v0.11.9 tool (https://www.bioinformatics.babraham.ac.uk/projects/fastqc/)^104^. To improve the quality of the raw data, TrimGalorev0.6.0 tool (https://www.bioinformatics.babraham.ac.uk/projects/trim_galore/)^105^ with ‘-q 30’ cutoff was employed to trim off the adapter sequences, uncalled and low-quality bases. Since the quality of raw reads obtained from the Set2 were not satisfactory, it was further filtered using BBMap v38 (https://jgi.doe.gov/data-and-tools/software-tools/bbtools/)^106^ with stringent parameters to address the problem associated with bad tile quality. The resulting clean reads obtained from both sequence sets were merged prior to assembly.

### De novo assembly and annotation

The merged clean data was subjected to denovo assembly using the default parameters of TRINITY v2.9.3 (http://trinityrnaseq.sourceforge.net/)^107^ with all parameters set to default. The input reads were aligned with the assembled transcriptome using Bowtie2 (https://bowtie-bio.sourceforge.net/bowtie2/)^108^ to assess the assembly quality. The assembled transcripts were preprocessed using SeqClean (https://sourceforge.net/projects/seqclean/files/) against NCBI Univec databases (ftp://ftp.ncbi.nih.gov/pub/UniVec/) with parameters ‘-l 200 -c 16’ and library-less repeat masking was carried out using RBR (http://www.ii.uib.no/~ketilbioinformatics/downloads/rbr)^109^ with parameters ‘--sparse-keys = 100’. TGICL (http://www.tigr.org/tdb/tgi/software/)^110^ was employed to cluster, reassemble and elongate the Trinity assembled transcripts. The assembled transcripts were then clustered to remove redundancy and produced unigenes which contained the longest transcript of each gene. The re-assembly was carried out with the default parameters. The assembled unigenes were functionally annotated by performing homology search against SwissProt, Pfam, GO and KEGG^111–114^ databases, using Trinotate v3.2.1 (https://github.com/Trinotate/Trinotate/releases)^115^ with a configuration file that was modified to use DIAMOND aligner (https://github.com/bbuchfink/diamond)^116^. The bar graph representing GO annotation results from Trinotate was obtained using WEGO portal (http://wego.genomics.org.cn/). For AMP annotation, the assembled unigenes were searched against with APD3^117^, CAMP_R3_^118^, and LAMP^119^ databases using BLASTx (e-value: 1^e−5^). The BLASTx outputs were then screened with a percentage identity of ≥ 80.

### Tissue specific expression profiling of AMP genes

The unigenes annotated as Avian Beta Defensin 2 (CL60147Contig), Avian Beta Defensin 13 (CL64604Contig1) and Cathelicidin 2 (CL31589Contig1) were selected for expression analysis. The expression profiles of the selected genes in 14 tissues of 3 crows were analyzed by real-time PCR using Roche -Light Cycler^®^ 480 System. About 1 µg of total RNA extracted from each tissue was reverse transcribed using Superscript™ III First-Strand System for RT-PCR (Invitrogen). Real-time PCR was performed in 10 µl reaction containing cDNA sample, SYBR green mix, gene-specific primers (Table 4), and double-distilled water. The expression level of the three genes were measured by the 2^(−ΔΔCt)^ method using β-actin as internal reference gene and oesophagus as calibrator tissue^56,120–122^.

**Table 4:** Primer sequences for real time PCR.

Gene name	Forward primer	Reverse primer
Avian beta-defensin 2 (AvBD-2)	ACAGCCATGAAGATCCTTTACC	GGCAAAGACAAACCTGGAGA
Avian beta-defensin 13 (AvBD-13)	CAGCAGTGCAGAAGCAACC	ATTGCTGCAGCTCCCTTC
Cathelicidin 2 (CATH-2)	CCGTGGATTCCTACAACCAG	TCCATCATGCTGAAGTTGAGTC
β- actin	CCCCACCTGAGCGTAAATACT	CCTGCTTGCTGATCCACAT

The statistical analysis of the data was carried out using multiple *t*-test analysis and one-way ANOVA. Data were repeated in triplicate and plotted with GraphPad Prism 10. Differences were defined as significant if P < 0.05.

### Characterization of *Corvus* CATH-2

Cathelicidin peptides have not yet been extensively studied in any of the crow species. In order to comprehend the mechanism of action of *Corvus* CATH-2, a member of cathelicidin family from *C. splendens*, we performed its structural and functional characterization.

#### Sequence analysis of CATH-2 peptide

The amino acid sequences of the peptide was predicted using the ExPASy-Translate tool (https://web.expasy.org/translate/) and was subjected to a homology search using BLASTP at NCBI. The related protein sequences from other avian species were retrieved from NCBI and multiple sequence alignment was performed using T-coffee server (https://tcoffee.crg.eu/)^123^. The presence of signal peptide in the translated AMP sequence was determined by using SignalP 6.0 program (https://services.healthtech.dtu.dk/services/SignalP-6.0/)^124^. The mature region was deduced by comparing the pro-peptide sequences and cleavage site of known CATH-2 peptides. EMBOSS PEPSTAT (https://www.ebi.ac.uk/Tools/seqstats/emboss_pepstats/) was used to analyze the physicochemical properties of the mature sequence^125^. The secondary structure was predicted by the SOPMA secondary structure prediction server (https://npsa-prabi.ibcp.fr/cgi-bin/npsa_automat.pl?page=/NPSA/npsa_sopma.html). The three dimensional structure of mature CATH-2 was generated using Quark ab initio structure prediction software (https://zhanggroup.org/QUARK/)^126^. The helical wheel diagram of the mature peptide was plotted with Heliquest software (http://heliquest.ipmc.cnrs.fr/cgi-bin/ComputParamsV2.py).

#### 3D structure prediction and MD simulation

To comprehend the molecular mechanism of antibacterial activity and toxicity of the peptide, we analyzed the interaction of CATH-2 with bacterial and mammalian membrane respectively using molecular dynamics. We performed MD simulation of CATH-2 using a mixture of palmitoyl-2-oleoyl-sn-glycero-3-phosphocholine (POPC) and palmitoyl-2-oleoyl-sn-glycero-3-phosphoglycerol (POPG) in a 2:1 ratio mimicking bacterial membrane and palmitoly-2-oleoyl-sn-glycero-3- phosphocholine alone mimicking mammalian membrane. The predicted 3D structure of the peptide was loaded onto the visual molecular dynamics (VMD) software and the system was embedded in a membrane environment containing POPC only and mixed POPC: POPG (2:1) with membrane dimensions of 57, 52 Å, membrane centre (x,y,z): − 2.013, − 1.516, − 34.785 respectively. The peptide was positioned on top of the lipid bilayer system in such a way that the distance from the centre of mass of the lipid layer to that of AMP’s center of mass was 30 Å. The system in the membrane environment was solvated in two steps, and the distance between the minimum and maximum z coordinates and the water box edges in the z-axis was fixed to 15 Å. The membrane system was solvated using water molecules (TIP3P model) which were positioned below and above the membrane plane with water box dimensions of 57.62, 55.28, 15.86 and 57.62, 55.28, 36.51 (x,y,z in Å) respectively and was neutralized using appropriate number of sodium and chloride ions. Molecular dynamics was carried out on this system using the NAMD molecular dynamics package^127^ and the CHARMM36 force field^128^. For all simulations, the time step of integration was selected to be 2 fs. The Equilibration steps were carried out in the NPT ensemble with the backbone restrained under explicit solvent, consisting of 1.00 ns of simulation. The temperature of 300 K was maintained using Langevin dynamics. The MD of the final systems was performed using Langevin dynamics with a temperature maintained at 300 K and consisted of 10.0 ns of simulation^129^.

#### Peptide synthesis

The mature peptide was synthesized by Fmoc solid-phase synthesis (SPPS) strategy using specific automated peptide synthesizer Autopep-001A^130^. The peptide was amidated at the C-terminus as previous research has shown that amidation at the C-terminal of the peptide is strongly linked to its stability^66^. To provide a C-terminus free carboxyl group of activated amino acid for strong and detachable covalent bonding, 4-(2′,4′-Dimethoxyphenyl-Fmoc-aminomethyl) phenoxy resin 100–200 mesh was used. Carboxylic group of N-terminal protected amino acid was activated and peptide amide coupling with the growing solid-phase system was initiated. Deprotection was performed using 20% piperidine in dimethylformamide. The final form of resin was washed with MeOH, CHCl3, and hexane, and dried. A combination of TFA (95%): TIS (2.5%): water (2.5%) was used to remove the peptide from polymer resin and side-chain protecting groups. The synthesized peptide was extracted from the solution using excess peroxide-free pure cold diethyl ether, which was subsequently rinsed seven times with diethyl ether prior to centrifugation to remove residual scavengers and protecting groups. The crude peptide was diluted in 5% acetonitrile solution, and the peptide purity was analyzed to be higher than 95% using reverse-phase HPLC on an RPC18 column (M/s Shimadzu Corporation, Japan). The molecular mass of the synthesized peptide was measured using ESI–MS.

#### Structural characterization using circular dichroism spectroscopy

To determine the secondary structure of the synthesized peptide, circular dichroism (CD) spectra were measured using a Jasco J-815 spectrophotometer at wavelengths ranging from 190 to 300 nm. 0.5 mg/ml of CATH2 peptide was prepared separately in water and 50 percent (v/v) trifluoroethanol (TFE)/water, and then applied to a quartz optical cell with path length of 2 mm at 25 °C. The spectra were measured from 190 to 300 nm and were averaged over three consecutive scans, and subsequently, the solvent’s CD signal was subtracted. Secondary structure prediction was calculated from the obtained CD spectra using the BeStSel analysis server (https://bestsel.elte.hu/)^131–133^.

#### Antimicrobial assay

Antimicrobial activity of the peptide against a few clinically important bacterial strains, namely *Escherichia coli* (MTCC 739), *Staphylococcus aureus* (MTCC 737), and *Bacillus cereus* (clinical isolate) was determined by a modified broth dilution assay as described previously^88^. The peptide’s minimum inhibitory concentration (MIC) was measured. The bacterial strains were cultured in fresh Mueller Hinton (MH) broth at 37 °C to mid-log phase and then diluted with MH broth to a final concentration of approximately 5 × 10^5^ CFU/ml. The peptide was subjected to serial dilutions in MH broth. Equal volumes (50 μl) of serially diluted concentrations of peptide and bacterial inoculums were poured to each well of a sterile 96-well microtitre plate. The positive and negative controls were broth with and without bacteria control, respectively. After incubation for 18 h at 37 °C, the optical density at 595 nm was determined using Enspire multimode plate reader from Perkin Elmer Inc. (Waltham, MA, USA). The assays were performed in triplicates with three biological replicates per experiment.

#### Haemolytic assay

The cytotoxicity of the synthesized peptide was measured using human erythrocytes^50,134,135^. Stored blood from the blood bank was used for the study. Erythrocytes were collected by the centrifugation of whole blood at 1000×*g* for 5 min at 4 °C. The collected erythrocytes were washed 3 times with PBS, and the precipitates were resuspended to 2% in PBS. Equal volumes (100 μl) of both erythrocytes suspension and twofold serial dilution of the synthetic peptide were added to 96 well plate. Positive and negative controls were erythrocytes suspended in Triton X-100 (0.1%) and PBS, respectively. After 1 h of incubation at 37 °C, the plate was centrifuged for 5 min at 1000×*g*. The supernatant was then transferred to a new plate and the haemoglobin release of was determined by monitoring the absorbance at 414 nm using Enspire Multimode plate reader from Perkin Elmer Inc. (Waltham, MA, USA). The assays were performed in triplicates with three biological replicates per experiment.

The following equation was used to calculate the percentage of hemolysis:

$$\% \;{\text{hemolysis}} = \left[ {\left( {{\text{A}}414\;{\text{nm}}\;{\text{in}}\;{\text{CATH-2}}- {\text{A}}414\;{\text{nm in PBS}}} \right)/\left( {{\text{A}}414\;{\text{nm}}\;{\text{in}}\;0.1\% \;{\text{Triton}}\;{\text{X-}}100 - {\text{A}}414\;{\text{nm}}\;{\text{in}}\;{\text{PBS}}} \right)} \right] \times 100\%$$

#### Molecular docking

The predicted 3D structure of CATH-2 peptide was subjected to docking simulation. The structure of the TLR4–MD-2 complex was retrieved from the Protein Data Bank (PDB ID: 2Z64) and the initial structure of TLR4–MD-2-*Corvus* CATH-2 was developed using Zdock3.0.2 (https://zdock.umassmed.edu/)^136^. Among the total decoy structures predicted by Zdock, the lowest energy structure in which CATH-2 bound to the LPS binding site of MD-2 was selected and used for the flexible docking analysis by RosettaDock (version3.5) (https://www.rosettacommons.org/docs/latest/application_documentation/docking/docking-protocol). Out of the 10,000 decoy structures generated by RosettaDock, the docking model with the least interfacial binding energy was chosen for subsequent analysis.

### Ethical approval

The animal studies were approved by Institutional Animal Ethics Committee (IAEC), College of Veterinary and Animal Sciences, Pookode, Kerala, India. (Approval number: IAEC/COVAS/PKD/I7/20I9). All possible efforts were made to replace, reduce, and refine animal experiments. The study complies with ARRIVE guidelines as well as ethical guidelines of Committee for the Purpose of Control and Supervision of Experiments on Animals (CPCSEA) formed by the Act of the Indian Parliament under the Prevention of Cruelty to Animals Act.

### Informed consent

As stored blood from the blood bank was used, the study does not require informed consent. Human Ethics Committee permission was obtained from Central University of Kerala, Kerala, India. (Approval number: CUK/IHEC/2019/060).

### Supplementary Information

Supplementary Table S1.

Supplementary Table S2.

Supplementary Table S3.

Supplementary Table S4.

## Data Availability

The datasets generated and analyzed during the current study are available in the NCBI BioProject database under accession number PRJNA591600.
